# Quantitative evaluations of vortex vein ampullae by adjusted 3D reverse projection model of ultra-widefield fundus images

**DOI:** 10.1038/s41598-021-88265-w

**Published:** 2021-04-26

**Authors:** Ryoh Funatsu, Hiroto Terasaki, Hideki Shiihara, Sumihiro Kawano, Mariko Hirokawa, Yasushi Tanabe, Tomoharu Fujiwara, Yoshinori Mitamura, Taiji Sakamoto, Shozo Sonoda

**Affiliations:** 1 Department of Ophthalmology, Kagoshima University Graduate School of Medical and Dental Sciences, Kagoshima, Japan; 2 Japan-Clinical Retina Study (J-CREST) Group, Kagoshima, Japan; 3 Department of Ophthalmology, Kurashiki Chuo Hospital, Kurashiki, Japan; 4 NIKON CORPORATION, Yokohama, Japan; 5 Department of Ophthalmology, Tokushima University Graduate School, Tokushima, Japan

**Keywords:** Medical research, Anatomy

## Abstract

The purpose of this study was to determine the number and location of vortex vein ampullae (VVA) in normal eyes. This was an observational retrospective study. Montage images of one on-axis and two off-axis ultra-widefield images of 74 healthy eyes were enhanced, and reverse projected onto a 3D model eye. The number and distance between the optic disc to each VVA in the four sectors were compared. The significance of correlations between these values and age, sex, visual acuity, refractive error, and axial length was determined. The mean number of VVA was 8.10/eye with 1.84, 2.12, 2.19 and 1.95 in upper lateral, lower lateral, upper nasal, and lower nasal sectors, respectively. The mean number of VVA/eye was significantly greater in men at 8.43 than women at 7.76 (*P* = 0.025). The mean distance between the optic disc and VVA was 14.15 mm, and it was 14.04, 15.55, 13.29 and 13.66 mm in the upper lateral, lower lateral, upper nasal and lower nasal sectors, respectively (all *P* < 0.05). The number and location of VVA can be obtained non-invasively, and the number was significantly higher in men than women. This technique can be used to determine whether these values are altered in a retinochoroidal disease.

## Introduction

The choroid plays an important role in the pathogenesis of various retinochoroidal diseases^1–3^. However, the function and structure of the choroid is not well understood mainly because of difficulties in observing and quantifying its characteristics in in situ images*.* Indocyanine green angiography (ICGA) has been a good method to image the choroidal vessels in situ, however its invasiveness prevents it from being widely used in healthy subjects^4^.

The necessity of knowing the entire choroidal circulation and structure is exemplified in the pachychoroid diseases. The pachychoroidal diseases are a major cause of vision reduction and is mainly present in older individuals worldwide^5,6^. The pathogenesis of these diseases has been investigated, and genetic factors and other factors were found to be significantly associated with them^7^. In addition, there have been several studies attributing alterations of the vascular structure of the choroid to pachychoroidal diseases^8–11^. Thus, Chung et al. reported that abnormal vascular perfusion of the choroid was associated with the development of choroidal neovascularizations in polypoidal choroidal vasculopathy^9^. Kishi et al. and our group reported that a large choroidal vessel was frequently present beneath the macula in eyes with central serous chorioretinopathy (CSC) which is consistent with the thicker choroid in the submacular area of eyes with CSC^10^.

To understand the relationship between the choroidal vascular structure and the function of the choroid and retina, it is necessary to determine the pattern of circulation of the entire choroid. Unfortunately, the vascular system of the entire choroid is not well known in both normal and diseased eyes. This is mainly due to the inability to obtain images of the entire extent of the choroid with no distortion for quantification. There was a recent report of a technique that uses local anesthesia and contact lenses to observe the entire choroid^12^, but the images obtained are in 2D making it difficult to quantify the distance and angle within the eye. A 3D projection method was developed in which the fundus photographs are reverse stereo projected onto a 3D eyeball model^13^. Although the angular information of these images were well preserved, this model cannot reflect the actual distance because of an inability to adjust for differences in the axial length of the eye. To overcome this limitation, we have developed a new model to determine the number and location of specific structures in the far peripheral areas of the ocular fundus.

Thus, the purpose of this study was to determine the number, distribution, and distances of the vortex vein ampullae (VVA) from the optic disc and fovea in normal human eyes in a non-invasive and quantitative manner. These findings will form the normative values to which findings from eyes with retinochoroidal diseases can be compared.

## Results

### Demographics of subjects

The mean age of all subjects was 36.53 ± 10.13 years with a range of 20 to 55 years. The mean BCVA was − 0.22 ± 0.08 logMAR units with a range of − 0.41 to − 0.19 logMAR units, the mean refractive error was − 3.26 ± 2.62 diopters (D) with a range of − 9.63 to − 0.75, and the axial length was 24.92 ± 1.11 mm with a range of 22.47 to 26.97. In the men, the mean age was 36.89 ± 10.35 years, the mean BCVA was − 0.21 ± 0.08 logMAR units, the mean refractive error was − 3.25 ± 2.43 D, and the mean axial length was 25.18 ± 1.11 mm. In the women, the mean age was 36.16 ± 10.06 years, the mean BCVA was − 0.22 ± 0.08 logMAR units, the mean refractive error was − 3.27 ± 2.83 D, and the mean axial length was 24.65 ± 1.06 mm. The men had significantly longer axial lengths than women, but the differences of the other parameters were not significant between men and women (Table 1).

**Table 1:** Patient demographics.

	All*	Males*	Females*	P value^§^
Sample size	74	37	37	–
Age (years)	36.53 ± 10.13	36.89 ± 10.35	36.16 ± 10.06	0.596
BCVA (logMAR)	− 0.22 ± 0.08	− 0.21 ± 0.08	− 0.22 ± 0.08	1.000
ES (D)	− 3.26 ± 2.62	− 3.25 ± 2.43	− 3.27 ± 2.83	0.844
AL (mm)	24.92 ± 1.11	25.18 ± 1.11	24.65 ± 1.06	0.053

*BCVA* best corrected visual acuity, *ES* equivalent spherical, *AL* axial length.

*Mean ± standard deviation.

^§^Mann–Whitney U test.

### Intraclass correlation coefficients of analytic method

The differences in the number of vortex vein ampullae on different days were not significant. The intraclass coefficients of the correlations for the intra-subject examinations at two time points by the same evaluator for the distance between the optic disc and vortex vein ampullae was 0.99, and that for the angle of the fovea—optic nerve head—vortex vein ampulla was 0.99 for all sectors. The intraclass correlation coefficient of the inter-rater examinations by the two evaluators for the distance was 0.96. The correlation of coefficient for the angle was more than 0.95 for all sectors. In addition, the intra-class correlation coefficient of the intra-rater at two time points on different days by one evaluator for the distance was 0.99. For the angle, it was 0.98 for all sectors (Supplementary Table S1).

### Differences in number of vortex vein ampullae for different imaging methods

We compared the number of vortex vein ampullae obtained by one on-axis image and the montage images of two off-axis images.The average number of vortex vein ampullae was 5.16 ± 1.19 in the on-axis images and 8.10 ± 1.44 in the montage images (*P* < 0.001**,** Mann–Whitney U test).

### Distribution of vortex vein ampullae

**Figure 1:**
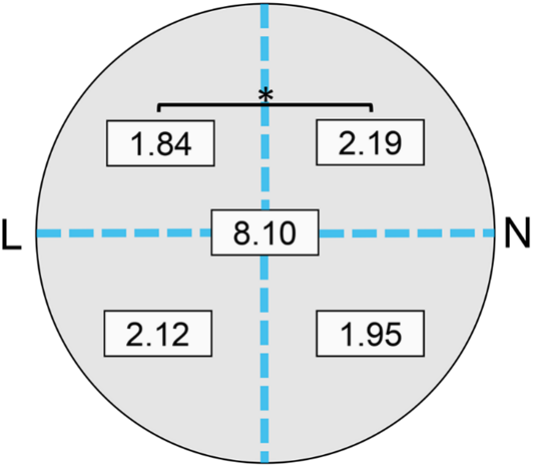
Distribution of number of vortex vein ampullae in four sectors in all subjects. The overall mean number of vortex vein ampullae (VVA) was 8.10/eye and 1.84, 2.12, 2.19 and 1.95 in upper lateral (UL), lower lateral (LL), upper nasal (UN) and lower nasal sector (LN), respectively. A statistically significant difference was observed between UL and UN sectors (*P = 0.012, Wilcoxon signed rank sum test) *L* lateral side, *N* nasal side.

Overall, there were 8.10 ± 1.44 VVA/eye. For the sectors, there were 3.96 ± 0.91 VVA/eye in the temporal sector, 4.14 ± 0.94 VVA/eye in the nasal sector, 4.03 ± 1.03 VVA/eye in the superior sector, and 4.07 ± 0.82 VVA/eye in the inferior sector. For the quadrants, there were 1.84 ± 0.66 VVA/eye in the upper temporal quadrant, 2.12 ± 0.64 VVA/eye in the lower temporal quadrant, 2.19 ± 0.72 VVA/eye in the upper nasal quadrant, and 1.95 ± 0.57 VVA/eye in the lower nasal quadrant (Supplementary Table S2). The differences in the number of VVA was significant among the four quadrants (*P* = 0.002, Friedman test), and a significant difference was observed between the upper temporal and the upper nasal quadrants (*P* = 0.012, Wilcoxon signed rank sum test, Fig. 1).

Simple regression analysis showed that there was a significant correlation between the number of vortex vein ampullae and the sex (*P* = 0.041, simple linear regression analysis, Table 2). When the factors associated with the number of vortex vein ampullae were selected by the backward forward stepwise method and multiple regression analysis was performed, a significant correlation was found only between the number of ampullae and sex (*P* = 0.041, backward forward stepwise method, Table 2).

**Table 2:** The correlation between the number of vortex vein ampulla per eye and age, gender, logMAR visual acuity, equivalent spherical, and axial length.

	Simple regression analysis		Multiple regression analysisa	
	Pearson correlation coefficient	P value	Pearson correlation coefficient	P value
Age	0.01	0.574	–	–
Gender	0.676	**0.041**	0.676	**0.041**
ES (D)	0.691	0.983	–	–
AL (mm)	0.01	0.819	–	–

*ES* equivalent spherical, *AL* axial length. These factors are selected by the backward forward stepwise method. Statistically significant results were written in bold letter.

### Sex differences in distribution of vortex vein ampullae

**Figure 2:**
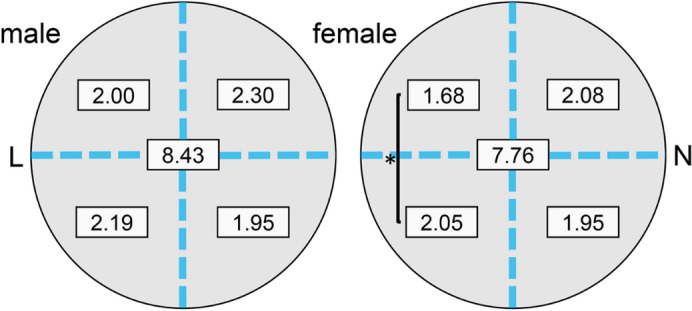
Distribution of vortex vein ampullae by sex. In men, the mean number of vortex vein ampullae (VVA) was 8.43/eye and 2.00, 2.19, 2.30 and 1.95 in the upper lateral (UL), lower lateral (LL), upper nasal (UN), and lower nasal sector (LN), respectively. In the women, the mean number of VVA was 7.76/eye and 1.68, 2.05, 2.08, and 1.95 in the upper lateral, lower lateral, upper nasal, and lower nasal sectors, respectively. A statistically significant difference was observed between UL and LN sectors in the women (*P = 0.043, Wilcoxon signed rank sum test) *L* lateral side, *N* nasal side.

In men, the overall mean number of VVA/eye was 8.43 ± 1.37, and the mean number by sectors was 4.19 ± 0.88 VVA/eye in the temporal sector, 4.23 ± 0.83 VVA/eye in the nasal sector, 4.30 ± 1.00 VVA/eye in the superior sector, and 4.14 ± 0.63 VVA/eye in the inferior sector. In addition, there were 2.00 ± 0.67 VVA/eye in the superior temporal sector, 2.19 ± 0.57 VVA/eye in the inferior temporal sector, 2.30 ± 0.66 VVA/eye in the superior nasal sector, and 1.95 ± 0.40 VVA/eye in the inferior nasal sector (Supplementary Table S3). There was a significant difference in the numbers of VVA/eye among the four sectors *(P* = 0.032, Friedman test), but the differences between each sector was not significant (Wilcoxon signed rank sum test, Fig. 2).

In women, the overall mean number of ampullae/eye was 7.76 ± 1.44. There were 3.73 ± 0.90 ampullae/eye in the temporal quadrant, 4.03 ± 1.04 ampullae/eye in the superior quadrant, 3.76 ± 0.01, ampullae/eye in the nasal quadrant, and 4.00 ± 0.97 ampullae/eye in the inferior quadrant. In addition, there was 1.68 ± 0.63 ampullae/eye in the superior temporal sector, 2.05 ± 0.70 ampullae/eye in the inferior temporal sector, 2.08 ± 0.76 ampullae/eye in the superior nasal sector, and 1.95 ± 0.70 ampullae/eye in the inferior nasal sector (Supplementary Table S3). There was a statistically significant difference among the four sectors (*P* = 0.027, Friedman test), and especially between the superior temporal sector and inferior temporal sectors (*P* = 0.043, Wilcoxon signed rank sum test, Fig. 2).

There was a significant difference in the number of VVA between men and women in the temporal, superior, and superior temporal sectors (*P* = 0.025, *P* = 0.044, *P* = 0.020, *P* = 0.048, respectively, Mann–Whitney U test, Supplementary Table S3).

### Distance of vortex vein ampullae from optic disc

**Figure 3:**
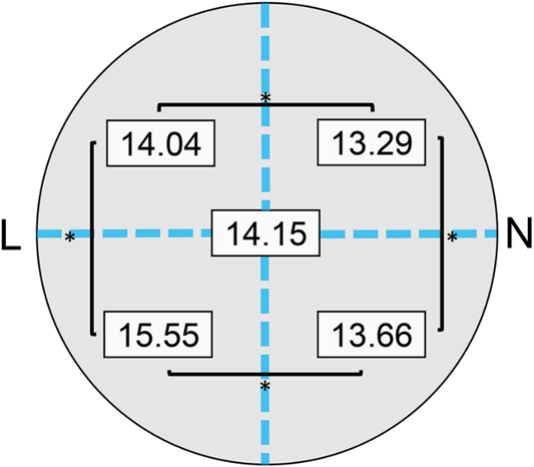
Location of vortex vein ampullae by sectors in all subjects. The mean distance between optic disc and vortex vein ampullae (VVA) was 14.15 mm for all eyes, and it was 14.04 mm, 15.55 mm, 13.29 mm, and 13.66 mm in the upper lateral (UL), lower lateral (LL), upper nasal (UN) and lower nasal (LN) sectors, respectively. A statistically significant difference was observed between all sectors (*P* = 0.038, upper nasal sector–lower nasal sector*, P* = 0.000, all pairs except for upper nasal sector–lower nasal sector, Wilcoxon signed rank sum test) *L* lateral side, *N* nasal side.

The overall mean distance between the optic disc and vortex vein ampulla was 14.15 ± 0.95 mm. The mean distance was 14.04 ± 1.12 mm in the superior temporal sector, 15.55 ± 1.31 mm in the inferior temporal sector, 13.29 ± 1.03 mm in the superior nasal sector, and 13.66 ± 1.20 mm in the inferior nasal sector (Supplementary Table S4). There was a statistically significant difference among the four sectors (*P* < 0.001, Friedman test), and for the quadrants, all pairs of sectors were also statistically different (*P* = 0.038, upper nasal sector—lower nasal sector*, P* < 0.001, all pairs except for upper nasal sector—lower nasal sector, Wilcoxon signed rank sum test, Fig. 3).

Calculations of the correlations between the mean distance of the optic disc and vortex vein ampullae and the age, axial length, sex, and refractive error showed that the axial length was the only factor that was significantly correlated to the distance *(P* < 0.001, simple linear regression backward forward stepwise method; Table 3).

**Table 3:** The correlation between the distance which is between vortex vein ampulla and optic disc per eye and age, gender, logMAR visual acuity, equivalent spherical, and axial length.

	Simple regression analysis		Multiple regression analysis^a^	
	Pearson correlation coefficient	P value	Pearson correlation coefficient	P value
Age	− 0.004	0.738	–	–
Gender	0.309	0.166	–	–
ES (D)	− 0.061	0.156	0.107	0.091
AL (mm)	0.434	** < 0.001**	0.600	** < 0.001**

*ES* equivalent spherical, *AL* axial length.

^a^These factors are selected by the backward forward stepwise method. Statistically significant results were written in bold letter.

## Discussion

There have been earlier studies that determined the number and location of the vortex vein ampullae. For example, Verma et al. used ICG angiography to examine 36 eyes with ages ranging from 21 to 79 years, and they reported that the mean number of vortex veins ampullae/eye is around 8 in healthy eyes of Caucasians^14^. Mori et al. studied 36 eyes and Takahashi et al. studied the fundus photographs of 25 eyes, and both reported the distribution of the vortex vein ampullae^15,16^. However, because the images were distorted and adjustments were not made for the size of each eye in comparison of our study, they were not able to quantify the location. Above all, the ICG examinations was too invasive to measure many normal individuals^4^.

As an alternative to ICGA, we analyzed the vortex veins in normal eyes by examining a montage of ultra-widefield fundus images. Even with the ultra-widefield images, only an average of 5.16 ± 1.19 vortex veins could be seen in single shot. Thus, a montage of multiple images was necessary to record all the vortex veins. We could not perform ICGA in current cases because this study was performed on healthy volunteers, and the use of ICGA was rejected by the Ethics Committee of our hospital. Therefore, it is not possible to compare the montage images and ICGA images. Earlier, we determined the agreement between the choroidal vasculature enhanced images, which used in this study, and the ICGA images of vortex veins in diseased eyes and reported that both images were comparable. The agreements did not vary with the ocular axial length, refractive error, and age^17^. In addition, we showed a high repeatability in our analysis as in the Supplementary Table S1. Thus, the present non-invasive analysis method can be used for the analysis of the vortex veins reliably.

To the best of our knowledge, our study is the first to examine the location and number of vortex vein ampullae in a non-invasive and quantifiable method with high reproducibility. The average number of vortex vein ampullae and distance from the optic disc to the ampullae are comparable to that reported earlier^14^. These findings strongly suggest that the methods we used were valid in terms of counting the number of vortex vein ampullae. Verma et al. used a stereo projection method to correct image distortions as in the present method^14^, but they did not correct for axial length in each case. On the other hand, the present method adjusted the ocular model for each axial length, and the measured distances had a significant positive correlation with the axial length (Table 3, Supplementary Table S5). Thus, the vortex vein analysis needed to be corrected for each ocular axial length as in our method. Our method which can analyze the vortex veins with correction for ocular axial length is the difference and novelty of this study from the report by Verma et al. Until now, the number and locations of the vortex vein ampullae have been reported only in Caucasians, Afro-Americans, and Hispanics^14^. Although the quantification method did not adjust for the differences in the axial lengths, the differences may not differ significantly between the different ethnic groups.

It was interesting to find that the number of vortex vein ampullae was significantly different in men than women. This difference was most obvious in the upper-temporal quadrant. There are two possible reasons in this result. First, there have been many reports that androgens activate the vascular endothelial cell growth factor and promote vascular endothelial cell recruitment and proliferation^18,19^. Anatomical and physiological investigations have shown structural and functional differences between men and women in the kidney microcirculatory system^20,21^. In the development of the choroidal vascular system, the vortex veins begins to grow around the eighth week of gestational age and are completed by around the sixteenth week^22^. From the viewpoint of the sex differences, masculinization begins around 8 weeks of gestational age, and the plasma concentration of male hormone is high between 8 and 17 weeks^23^. Given that androgens affect angiogenesis, and that the vascularity system of the different organs varies between the sexes, our present findings may be reasonable.

**Figure 4:**
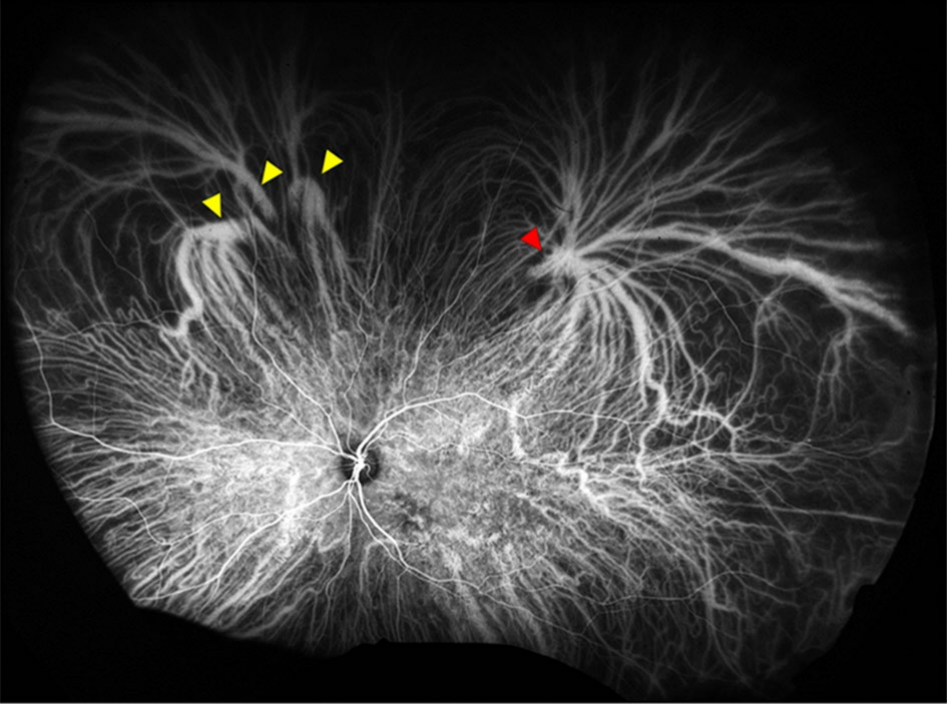
Image of indocyanine green fundus angiogram of the left eye taken by Optos California in an early phase. The differences in the forms of the vortex vein ampulla between upper lateral sector (red arrowhead) and upper nasal sector (yellow arrowhead). The vortex veins converged after passing through the scleral surface in the upper nasal sector, on the other hand, they converge before entering the sclera in the upper lateral sector.

Second, the vortex veins pass through the sclera to carry blood out of the eye^24^. The sclera is mainly composed of rigid connective tissue which is composed of interwoven chondroitin sulfate, dermatan sulfate, hyaluronic acid, and collagen fibers. These compound are influenced by sex hormones^25,26^. The sclera plays a role in supporting the path of the vortex veins, and structural differences in the sclera are thought to affect the choroidal hemodynamics^24,27^. For example, vortex veins may merge within the eye before penetrating the sclera, or they may merge within the sclera (Fig. 4)^24^. In the former case, it was judged as one vortex vein ampullae in this study, and in the latter case, it is judged as two. The difference in the way of merging of the vortex veins between men and women may have contributed to these findings although this study could not detect these differences.

Vortex veins are thought to be involved in the pathogenesis of diseases such as polypoidal choroidal vasculopathy, central serous chorioretinopathy, and uveal effusion^1–3,28^. The present results are the normative data that should help in understanding the pathophysiological changes in these disorders.

There are several limitations in this study. The retrospective nature, comparatively small sample size, and single center design indicate that the sampling of the subjects was not completely free from bias. In addition, there is a limitation of this algorism and the 3D eyeball model because we assumed that the examined eye was a regular sphere. In fact, the eye is not always a perfect sphere, and this model is not applicable to every eye such as the one with staphylomas. However, it is useful for most eyes to be analyzed for the locations regions because this model can reveal the angle, distance, and the area of ocular fundus at the same time accurately with high reproducibility and less invasiveness. Finally, the Japanese population is known to be the most myopic population in the world^29^. As a result, most of the subjects were myopic. Although the axial length could be corrected for in our analysis by our method, this may have affected the results. On the other hand, the reliability of the examination was high because no pathological factors such as cataract or vitreous opacities were present in healthy individuals and the understanding of the examination was high in the present examinees.

In conclusion, the results showed that our method can construct accurate images of the vascular pattern of the choroid in normal eyes from the central area to the far periphery noninvasively. With this method, the number, distribution, and distance from the optic disc of the vortex veins ampullae could be quantified with good inter- and intraclass correlations. The values found can be considered the normative values to which values from diseased eyes can be compared.

## Methods

This was a retrospective study of 37 normal men and 37 normal women who volunteered. The procedures used were approved by the Ethics Committee of the Kagoshima University, Kagoshima, Japan (no. 16012) and Tokushima University, Tokushima, Japan (no. 2334). This study was registered in the University Hospital Medical Network clinical trials registry in November 2013 as “UMIN000012310, Choroidal Structure on Images for Healthy Eyes”. All the procedures conformed to the tenets of the Declaration of Helsinki, and all participants signed an informed consent form.

### Subjects and exclusion criteria

This study enlisted 95 volunteers from the Kagoshima University Hospital or the Tokushima University Hospital from April 2018 to September 2019. Of these, 37 men and 37 women met the inclusion criteria. After an initial examination, those with known eye diseases, those with axial length longer than 27 mm, or those whose widefield fundus images were blurry were excluded.

### Eyeball model and axial length adjustments for accurate analysis

There are problems in analyzing the location of the VVA in the Optos California retinal imaging device (Optos PLC, Edinburgh, UK). In this device, the image of the three-dimensional eye is displayed in two-dimensional fundus photographs. Care must then be taken in quantifying the distances, angles, and areas of the structures because they appear to be the shortest distance in the two-dimensional image which is different from that of the actual eye. In addition, the axial length is fixed at 24 mm in measurements made in current available ocular image analyzers, e.g., Optos Advance^13^. However, different results are obtained by using different axial lengths in spherical bodies of different sizes (Supplementary Table S5).

To overcome these problems, we used reverse stereo projection method and axial-length adjustable software in this study. The methods are explained in detail as follows.

#### Eyeball model

In the Optos California retinal imaging device (Optos PLC, Edinburgh, UK), the image of the three-dimensional eye is displayed in two dimensional fundus photographs. Care must then be taken in quantifying the distances, angles, and areas of the structures because they appear to be the shortest distance in the two-dimensional image which is different from that of the actual eye (Supplementary Fig. S1).

To resolve this problem, we projected the 2D fundus image onto a three dimensional (3D) eyeball by reverse stereo projection so that the measurements on the actual eye can be performed more accurately^30,31^ (see Supplementary Video S1).

In addition, the distance, angle, and the area of the fundus structures could be measured accurately after it was projected onto a sphere. The spherical triangulation method, which is a commonly used method of measurement, was used^30^.

#### Influence of axial length

Although the eye is not a perfect sphere, it is assumed to be perfect sphere for the measurements. In general, the axial length is fixed at 24 mm in measurements made in ocular models, e.g., Optos advance^13^. However, different results are obtained by using different axial lengths in spherical bodies of different sizes. Thus, if the actual axial length is 22 mm but is assumed to be 24 mm, the distance from the optic disc to the vortex vein, which is around 14 mm, will be estimated to be 1 mm longer (Supplementary Table S5). To overcome this kind of discrepancy, we used a model in which the size of the spherical body can be changed for each axial length.

The distance from the optic disc to the vortex vein ampulla was measured. In addition, the angle formed at the intersection of lines running from the fovea to the vortex vein ampulla and a line running from the center of the optic disc to the ampulla was calculated. The line connecting the fovea and the center of the optic disc was defined as the horizontal axis, and the straight line perpendicular to the horizontal line was defined as the meridian or vertical axis. These two axes were used to divide the montage image into four sectors, viz., the upper lateral (UL), lower lateral (LL), upper nasal (UN), and lower nasal (LN) sectors.

### Montage of ultra-widefield images by conformal projection

**Figure 5:**
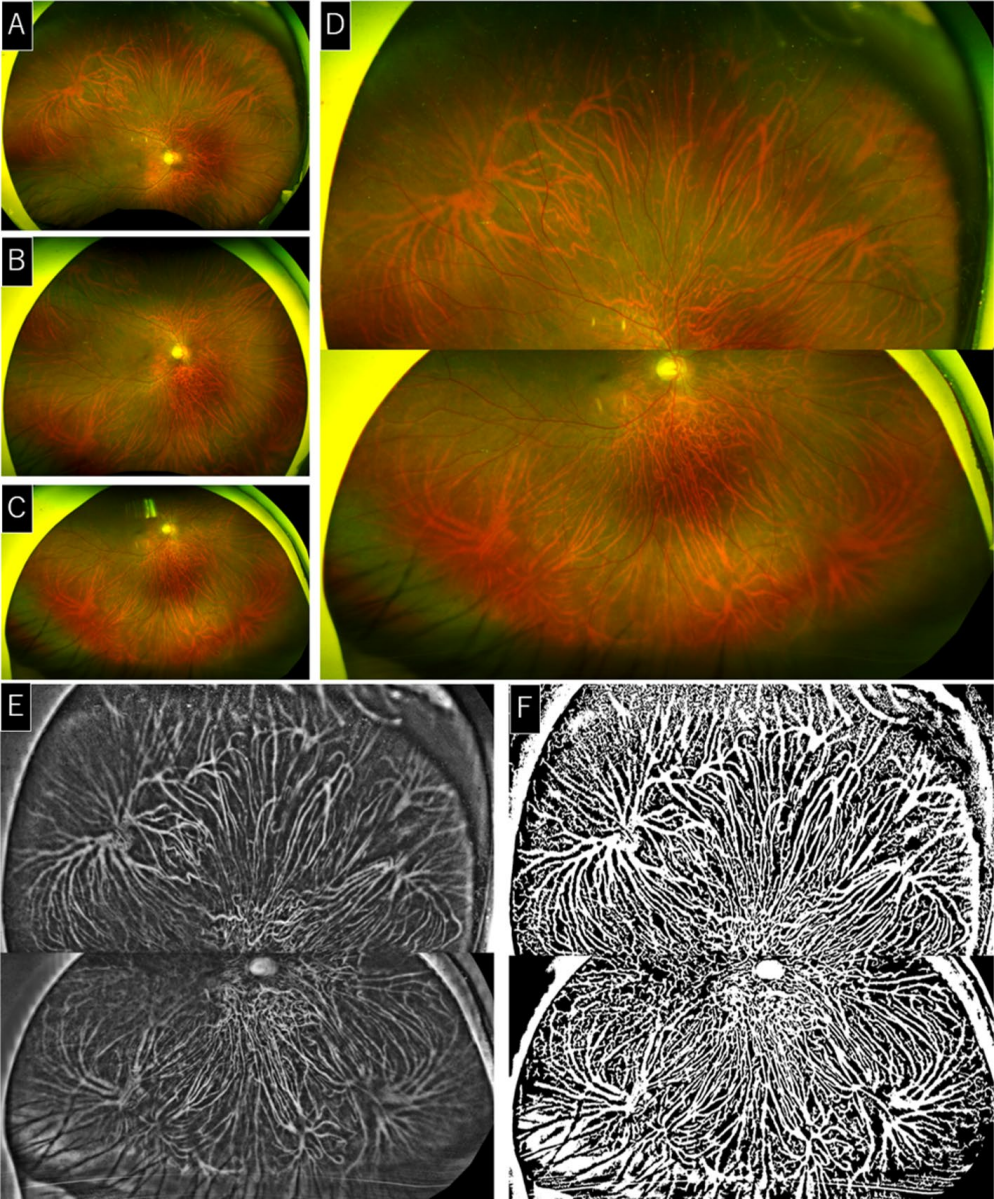
Montage of ultra-widefield images by conformal projection. One on-axis image and two off-axis images were acquired. (**A**–**C**). The images underwent geometric transformation to obtain a wider fundus photograph (**D**). The acquired montage images were processed by binarization of choroidal vessels using our previous method (**E**,**F**).

The entire extent of the vortex veins cannot be viewed in the frontal images of ultra-widefield fundus images taken with Optos (Fig. 5A–C). To observe the full extent of the vortex veins, it is necessary to acquire Optos images that include the periphery of the fundus by changing the viewing angle in two directions (Fig. 5A,C). An accurate montage of multiple images allowed us to obtain an image that displayed the entire vortex veins (Fig. 5D).To photograph the entire extent of the fundus, one on-axis image and two off-axis images were acquired with the Optos California device. For the off-axis photographs, the subject was asked to fixate upward or downward. Then, a geometric transformation of the three images was performed using the Optos Advance software (Optos PLC)^13^.

The off-axis images were geometrically transformed to conform to the on-axis image, and a montage was constructed. Geometric transformation is a common technique used in performing alignment^31^. When the horizontal direction of the fundus photograph was set to 0°, auxiliary lines were drawn in the directions of − 10°, 30°, 70°, 110°, 150°, and 190°, and the fundus was divided into five regions. Then, pairs were made for the frontal and upward views, and frontal and downward views, and the optic disc of each pair and the five vascular bifurcations of the five fundus regions, a total of 12 points, were aligned and combined to minimize the distortions.

In this way, an image of approximately the entire extent of the retina was obtained. To view the choroidal vessels clearly, the acquired montage image was subjected to enhancement processing using a method we have described in detail (Fig. 5E,F)^28^.

The distance from the optic disc to the VVA was calculated. In addition, the angle formed at the intersection of lines running from the fovea to the ampulla and a line running from the optic disc to the ampulla was calculated. The line connecting the fovea and the optic disc was defined as the horizontal axis, and the straight line perpendicular to the horizontal line and the optic disc was defined as the meridian or vertical axis.

### Evaluations of vortex vein ampullae

The center of the ampulla was taken to be the point of intersection of the straight lines drawn through the thicker blood vessels flowing into the ampulla (Supplementary Fig. S2). The number of VVA was compared using the on-axis and the montage images. The number of VVA/eye was compared for each of the four sectors of the fundus and compared between men and women. The relationships between the number of VVA and the sex, age, visual acuity, refractive error (spherical equivalent), and axial length were calculated. Similarly, the distance between the optic disc and the VVA was determined and compared for each sector. In addition, the correlations between the distance and the sex, age, BCVA, refractive error, and axial length were calculated.

### Intraclass correlation coefficients of analytic method

The images were evaluated by two masked evaluators. To determine the intraclass correlation coefficients of one evaluator, the evaluator selected the images of 10 randomly selected subjects and analyzed the images. The intraclass correlation coefficients of two evaluators was determined from their analyses of the images of the same subjects. The intraclass correlation coefficient of all subjects was also analyzed by one evaluator on two different dates and times to calculate the intra-rater intraclass coefficients of correlation.

### Statistical analyses

The significance of the differences in the number of vortex vein ampullae in the on-axis images and the montage images was determined by Mann–Whitney U tests. The significance of the differences in the average number of ampullae and the differences in the distance between the optic nerve head and the ampullae were determined by Friedman tests and Wilcoxon signed rank sum tests. The factors significantly correlated to the numbers and distances were examined by simple regression analysis, and the factors examined were selected by the backward-forward stepwise method and examined by multiple regression analysis. Differences in the number and distribution of venous vein ampullae in each sector were stratified by sex, and examined by the Friedman tests, Wilcoxon signed rank sum tests, and Mann–Whitney U tests. In addition, multiple tests were performed with the Holm correction. The R statistical software (version 3.6.1) was used for the statistical analyses.

## Supplementary Information

Supplementary Table S1.

Supplementary Table S2.

Supplementary Table S3.

Supplementary Table S4.

Supplementary Table S5.

Supplementary Figure S1.

Supplementary Figure S21.

Supplementary Video S1.
