# Supplementary material for: Quantitative evaluations of vortex vein ampullae by adjusted 3D reverse projection model of ultra-widefield fundus images
Source: Sci Rep. 2021 Apr 26;11:8916. doi: 10.1038/s41598-021-88265-w (PMC8076294; doi:10.1038/s41598-021-88265-w)
Supplement: Supplementary file 1 — Supplementary Table S1. [file 41598_2021_88265_MOESM1_ESM.docx]

**Quantitative evaluations of vortex vein ampullae by adjusted**

**3D reverse projection model of ultra-widefield fundus images**

Ryoh Funatsu^1,2^, Hiroto Terasaki^1,2^, Hideki Shiihara^1,2^, Sumihiro Kawano^3^, Mariko Hirokawa^4^, Yasushi Tanabe^4^, Tomoharu Fujiwara^4^, Yoshinori Mitamura^2,5^, Taiji Sakamoto^1,2^, Shozo Sonoda^1,2^

^1^Department of Ophthalmology, Kagoshima University Graduate School of Medical and Dental Sciences, Kagoshima, Japan.

^2^Japan-Clinical Retina Study (J-CREST) group, Kagoshima, Japan

^3^Department of Ophthalmology, Kurashiki chuo hospital, Kurashiki, Japan

^4^NIKON CORPORATION

^5^Department of Ophthalmology, Tokushima University Graduate School, Tokushima, Japan

**Supplementary Table S1**

**Intraclass correlation coefficient of the analytic method^*^**

|  | Intra-subject | Inter-rater | Intra-rater |
| --- | --- | --- | --- |
| distance^§^ |  |  |  |
| Whole eye | 0.99 (0.98 – 0.99) | 0.96 (0.95 – 0.96) | 0.99 (0.98 – 0.99) |
| angle^§§^ |  |  |  |
| Upper lateral side | 0.99 (0.97 – 1.00) | 0.95 (0.94 – 0.97) | 0.98 (0.97 – 0.98) |
| Lower lateral side | 0.99 (0.96 – 1.00) | 0.96 (0.95 – 0.97) | 0.98 (0.98 – 0.99) |
| Upper nose side | 0.99 (0.96 – 1.00) | 0.98 (0.97 – 0.98) | 0.99 (0.98 – 0.99) |
| Lower nose side | 0.99 (0.97 – 1.00) | 0.97 (0.96 – 0.98) | 0.98 (0.97 – 0.98) |
| ^*^: intraclass correlation coefficient (95 % confidence interval)  ^§^: the distance between optic disc and vortex vein ampulla  ^§§^: the angle of fovea - optic nerve head - vortex vein ampulla | | | |
