# Supplementary material for: Quantitative evaluations of vortex vein ampullae by adjusted 3D reverse projection model of ultra-widefield fundus images
Source: Sci Rep. 2021 Apr 26;11:8916. doi: 10.1038/s41598-021-88265-w (PMC8076294; doi:10.1038/s41598-021-88265-w)
Supplement: Supplementary file 2 — Supplementary Table S2. [file 41598_2021_88265_MOESM2_ESM.docx]

**Quantitative evaluations of vortex vein ampullae by adjusted**

**3D reverse projection model of ultra-widefield fundus images**

Ryoh Funatsu^1,2^, Hiroto Terasaki^1,2^, Hideki Shiihara^1,2^, Sumihiro Kawano^3^, Mariko Hirokawa^4^, Yasushi Tanabe^4^, Tomoharu Fujiwara^4^, Yoshinori Mitamura^2,5^, Taiji Sakamoto^1,2^, Shozo Sonoda^1,2^

^1^Department of Ophthalmology, Kagoshima University Graduate School of Medical and Dental Sciences, Kagoshima, Japan.

^2^Japan-Clinical Retina Study (J-CREST) group, Kagoshima, Japan

^3^Department of Ophthalmology, Kurashiki chuo hospital, Kurashiki, Japan

^4^NIKON CORPORATION

^5^Department of Ophthalmology, Tokushima University Graduate School, Tokushima, Japan

**Supplementary Table S2**

**The mean number of vortex vein ampulla**

|  | Mean ± SD (min. – max.) |
| --- | --- |
| Whole eye | 8.10 ± 1.44 (5 - 12) |
|  |  |
|  |  |
| Lateral | 3.96 ± 0.91 (2 - 6) |
| Nasal | 4.14 ± 0.94 (2 - 7) |
|  |  |
|  |  |
| Upper | 4.03 ± 1.03 (2 - 6) |
| Lower | 4.07 ± 0.82 (3 - 6) |
|  |  |
|  |  |
| Upper lateral | 1.84 ± 0.66 (1 - 3) |
| Lower lateral | 2.12 ± 0.64 (1 - 4) |
| Upper nasal | 2.19 ± 0.72 (1 - 4) |
| Lower nasal | 1.95 ± 0.57 (1 - 4) |
| SD: standard deviation, min.: minimum,  max: maximum | |
