# Supplementary material for: Quantitative evaluations of vortex vein ampullae by adjusted 3D reverse projection model of ultra-widefield fundus images
Source: Sci Rep. 2021 Apr 26;11:8916. doi: 10.1038/s41598-021-88265-w (PMC8076294; doi:10.1038/s41598-021-88265-w)
Supplement: Supplementary file 3 — Supplementary Table S3. [file 41598_2021_88265_MOESM3_ESM.docx]

**Quantitative evaluations of vortex vein ampullae by adjusted**

**3D reverse projection model of ultra-widefield fundus images**

Ryoh Funatsu^1,2^, Hiroto Terasaki^1,2^, Hideki Shiihara^1,2^, Sumihiro Kawano^3^, Mariko Hirokawa^4^, Yasushi Tanabe^4^, Tomoharu Fujiwara^4^, Yoshinori Mitamura^2,5^, Taiji Sakamoto^1,2^, Shozo Sonoda^1,2^

^1^Department of Ophthalmology, Kagoshima University Graduate School of Medical and Dental Sciences, Kagoshima, Japan.

^2^Japan-Clinical Retina Study (J-CREST) group, Kagoshima, Japan

^3^Department of Ophthalmology, Kurashiki chuo hospital, Kurashiki, Japan

^4^NIKON CORPORATION

^5^Department of Ophthalmology, Tokushima University Graduate School, Tokushima, Japan

**Supplementary Table S3**

**The comparison the distribution of vortex vein ampulla between male and female**

|  | male* |  | Female* | p value^§^ |
| --- | --- | --- | --- | --- |
| whole eye | 8.43 ± 1.37 (5 - 11) |  | 7.76 ± 1.44 (5 - 12) | **0.025** |
|  |  |  |  |  |
|  |  |  |  |  |
| Lateral | 4.19 ± 0.88 (3 - 6) |  | 3.73 ± 0.90 (2 - 6) | **0.044** |
| Nasal | 4.23 ± 0.83 (2 - 6) |  | 4.03 ± 1.04 (2 - 7) | 0.294 |
|  |  |  |  |  |
|  |  |  |  |  |
| Upper | 4.30 ± 1.00 (2 - 6) |  | 3.76 ± 1.01 (2 - 6) | **0.020** |
| Lower | 4.14 ± 0.63 (3 - 5) |  | 4.00 ± 0.97 (3 - 6) | 0.356 |
|  |  |  |  |  |
|  |  |  |  |  |
| Upper lateral | 2.00 ± 0.67 (1 - 3) |  | 1.68 ± 0.63 (1 - 3) | **0.048** |
| Lower lateral | 2.19 ± 0.57 (1 - 3) |  | 2.05 ± 0.70 (1 - 4) | 0.299 |
| Upper nasal | 2.30 ± 0.66 (1 - 4) |  | 2.08 ± 0.76 (1 - 4) | 0.185 |
| Lower nasal | 1.95 ± 0.40 (1 - 3) |  | 1.95 ± 0.70 (1 - 4) | 0.699 |
| *: mean ± Standard Deviation (min. – max.)  ^§^: Mann - Whitney U test | | | | |
