# Supplementary material for: Quantitative evaluations of vortex vein ampullae by adjusted 3D reverse projection model of ultra-widefield fundus images
Source: Sci Rep. 2021 Apr 26;11:8916. doi: 10.1038/s41598-021-88265-w (PMC8076294; doi:10.1038/s41598-021-88265-w)
Supplement: Supplementary file 4 — Supplementary Table S4. [file 41598_2021_88265_MOESM4_ESM.docx]

**Quantitative evaluations of vortex vein ampullae by adjusted**

**3D reverse projection model of ultra-widefield fundus images**

Ryoh Funatsu^1,2^, Hiroto Terasaki^1,2^, Hideki Shiihara^1,2^, Sumihiro Kawano^3^, Mariko Hirokawa^4^, Yasushi Tanabe^4^, Tomoharu Fujiwara^4^, Yoshinori Mitamura^2,5^, Taiji Sakamoto^1,2^, Shozo Sonoda^1,2^

^1^Department of Ophthalmology, Kagoshima University Graduate School of Medical and Dental Sciences, Kagoshima, Japan.

^2^Japan-Clinical Retina Study (J-CREST) group, Kagoshima, Japan

^3^Department of Ophthalmology, Kurashiki chuo hospital, Kurashiki, Japan

^4^NIKON CORPORATION

^5^Department of Ophthalmology, Tokushima University Graduate School, Tokushima, Japan

**Supplementary Table S4**

**The mean distance between optic disc and vortex vein ampulla**

|  | mean ± SD (min. – max.) |
| --- | --- |
| Whole eye | 14.15 ± 0.95 (11.24 – 16.44) |
|  |  |
|  |  |
| Upper lateral | 14.04 ± 1.12 (11.60 – 16.75) |
| Lower lateral | 15.55 ± 1.31 (11.95 – 18.90) |
| Upper nasal | 13.29 ± 1.03 (10.03 – 15.00) |
| Lower nasal | 13.66 ± 1.20 (10.70 – 16.80) |
| SD: standard deviation | |
