# Supplementary material for: Quantitative evaluations of vortex vein ampullae by adjusted 3D reverse projection model of ultra-widefield fundus images
Source: Sci Rep. 2021 Apr 26;11:8916. doi: 10.1038/s41598-021-88265-w (PMC8076294; doi:10.1038/s41598-021-88265-w)
Supplement: Supplementary file 5 — Supplementary Table S5. [file 41598_2021_88265_MOESM5_ESM.docx]

**Quantitative evaluations of vortex vein ampullae by adjusted**

**3D reverse projection model of ultra-widefield fundus images**

Ryoh Funatsu^1,2^, Hiroto Terasaki^1,2^, Hideki Shiihara^1,2^, Sumihiro Kawano^3^, Mariko Hirokawa^4^, Yasushi Tanabe^4^, Tomoharu Fujiwara^4^, Yoshinori Mitamura^2,5^, Taiji Sakamoto^1,2^, Shozo Sonoda^1,2^

^1^Department of Ophthalmology, Kagoshima University Graduate School of Medical and Dental Sciences, Kagoshima, Japan.

^2^Japan-Clinical Retina Study (J-CREST) group, Kagoshima, Japan

^3^Department of Ophthalmology, Kurashiki chuo hospital, Kurashiki, Japan

^4^NIKON CORPORATION

^5^Department of Ophthalmology, Tokushima University Graduate School, Tokushima, Japan

**Supplementary Table S5**

**The simulation case of utilizing 3D eye model which can be adjusted by axial length**

| Axial length  of 3D eye model |  | The distance between  a vortex vein and optic disc | Difference from the original 3D eye model |
| --- | --- | --- | --- |
| +3 mm |  | 15.85 mm | + 1.75 |
| +2 mm |  | 15.26 mm | + 1.16 |
| +1 mm |  | 14.68 mm | + 0.58 |
| 24.21 mm  (Original) |  | 14.10 mm | - |
| -1 mm |  | 13.52 mm | -0.58 |
| -2 mm |  | 12.94 mm | -1.16 |
| -3 mm |  | 12.35 mm | - 1.75 |
