# Supplementary material for: Quantitative evaluations of vortex vein ampullae by adjusted 3D reverse projection model of ultra-widefield fundus images
Source: Sci Rep. 2021 Apr 26;11:8916. doi: 10.1038/s41598-021-88265-w (PMC8076294; doi:10.1038/s41598-021-88265-w)
Supplement: Supplementary file 6 — Supplementary Figure S1. [file 41598_2021_88265_MOESM6_ESM.docx]

**Quantitative evaluations of vortex vein ampullae by adjusted**

**3D reverse projection model of ultra-widefield fundus images**

Ryoh Funatsu^1,2^, Hiroto Terasaki^1,2^, Hideki Shiihara^1,2^, Sumihiro Kawano^3^, Mariko Hirokawa^4^, Yasushi Tanabe^4^, Tomoharu Fujiwara^4^, Yoshinori Mitamura^2,5^, Taiji Sakamoto^1,2^, Shozo Sonoda^1,2^

^1^Department of Ophthalmology, Kagoshima University Graduate School of Medical and Dental Sciences, Kagoshima, Japan.

^2^Japan-Clinical Retina Study (J-CREST) group, Kagoshima, Japan

^3^Department of Ophthalmology, Kurashiki chuo hospital, Kurashiki, Japan

^4^NIKON CORPORATION

^5^Department of Ophthalmology, Tokushima University Graduate School, Tokushima, Japan

**Supplementary Figure S1**


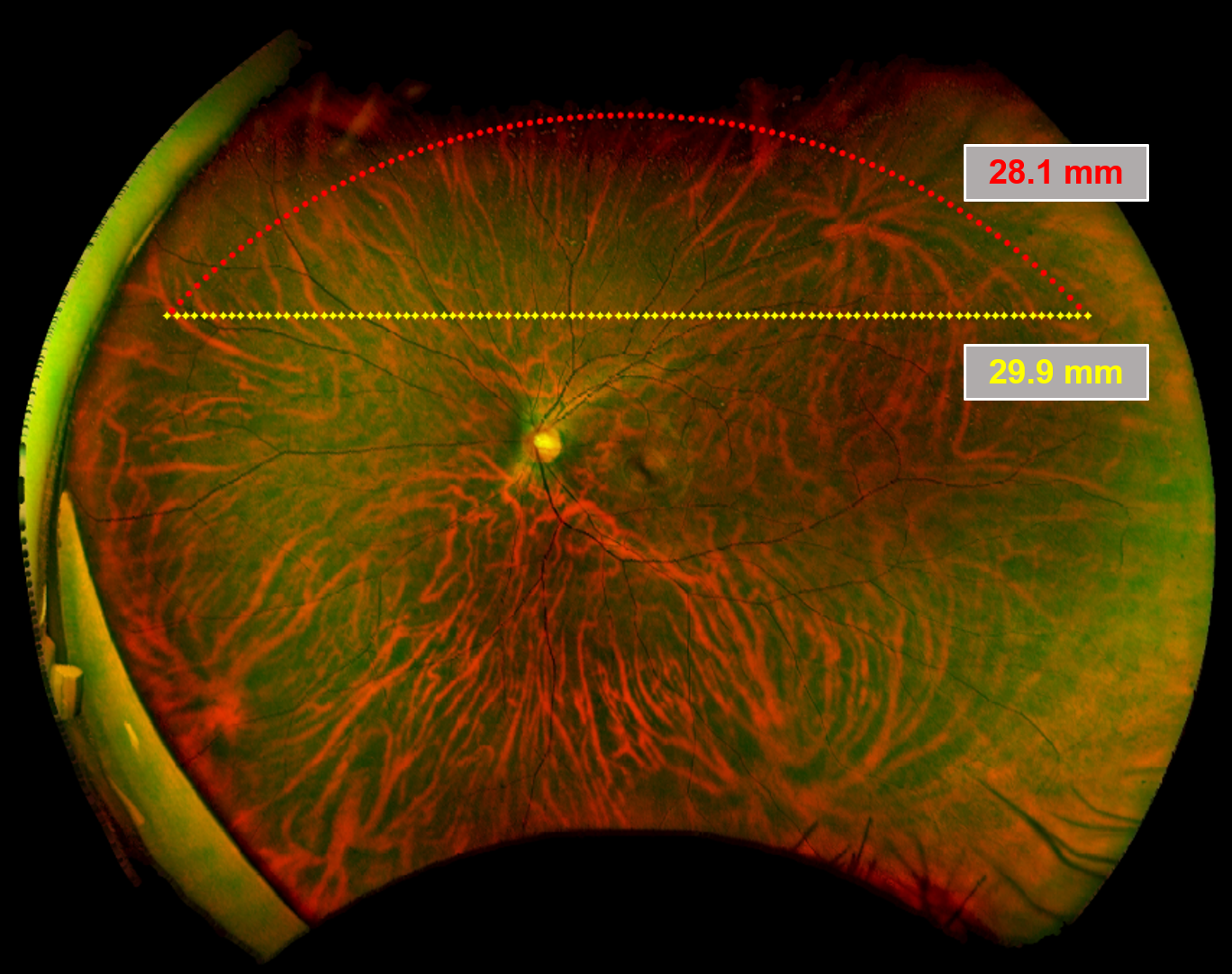


**Quantifying the correct distances in 2D wide-field image.**

The dotted yellow line represents the minimum distance on the fundus photograph and the dotted red line represents the actual minimum distance on the eyeball model. The dotted yellow line represents the minimum distance on the 2D fundus photograph and the dotted red line represents the actual minimum distance on the 3D eyeball model. The calculated distance of the red and yellow dotted lines on the surface of the eye are 28.1 mm and 29.9 mm, respectively.
