# Supplementary material for: Quantitative evaluations of vortex vein ampullae by adjusted 3D reverse projection model of ultra-widefield fundus images
Source: Sci Rep. 2021 Apr 26;11:8916. doi: 10.1038/s41598-021-88265-w (PMC8076294; doi:10.1038/s41598-021-88265-w)
Supplement: Supplementary file 7 — Supplementary Figure S21. [file 41598_2021_88265_MOESM7_ESM.docx]

**Quantitative evaluations of vortex vein ampullae by adjusted**

**3D reverse projection model of ultra-widefield fundus images**

Ryoh Funatsu^1,2^, Hiroto Terasaki^1,2^, Hideki Shiihara^1,2^, Sumihiro Kawano^3^, Mariko Hirokawa^4^, Yasushi Tanabe^4^, Tomoharu Fujiwara^4^, Yoshinori Mitamura^2,5^, Taiji Sakamoto^1,2^, Shozo Sonoda^1,2^

^1^Department of Ophthalmology, Kagoshima University Graduate School of Medical and Dental Sciences, Kagoshima, Japan.

^2^Japan-Clinical Retina Study (J-CREST) group, Kagoshima, Japan

^3^Department of Ophthalmology, Kurashiki chuo hospital, Kurashiki, Japan

^4^NIKON CORPORATION

^5^Department of Ophthalmology, Tokushima University Graduate School, Tokushima, Japan

**Supplementary Figure S2**

**
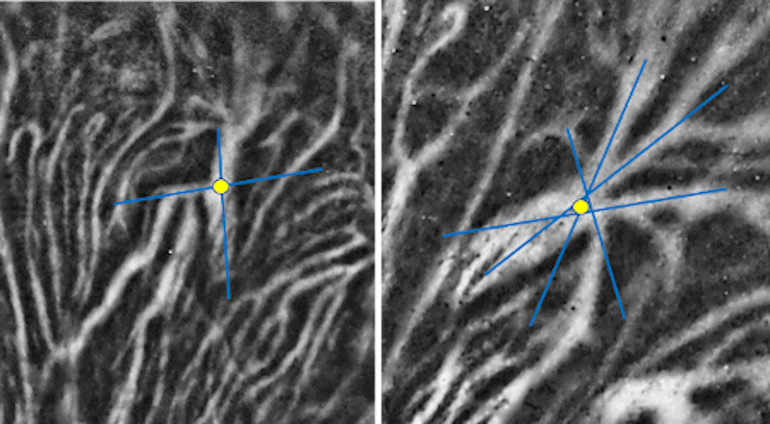
**

**Representative images of vortex vein ampullae with the marker of the location.** The intersection or the center (yellow circle) formed by the straight line (blue line) drawn from the thick blood vessel is defined as the location of the vortex vein ampulla.
